# Initial experience for first pass cardiac perfusion with iterative reconstruction in patients

**DOI:** 10.1186/1532-429X-17-S1-Q109

**Published:** 2015-02-03

**Authors:** Laura Iacuzio, Stéphane Rusek, Solenne Tutenuit, Michael O Zenge, Christoph Forman, Michaela Schmidt, Karen Mkhitaryan

**Affiliations:** 1 MR service, Centre Cardio Thoracique Monaco, Monte Carlo, Monaco; 2 Siemens AG, Erlangen, Germany

## Background

MR stress perfusion is a non-invasive, reliable and safe test for ischemic heart disease [1]. Recent publications reported sensitivity and specificity of 89% and 80% respectively [2]. Image quality improvements seem to be essential for improving the predictive value of the method. This leads to the dilemma of finding a compromise between high spatial resolution and sufficient SNR. Lately iterative reconstruction demonstrated great promise in improving SNR [3]. The aim of the current study was to compare cardiac perfusion in 24 patients reconstructed with product and a novel prototype iterative reconstruction.

## Methods

24 patients (mean age 62 ±15) were examined on a 1.5T clinical MR scanner (MAGNETOM Aera, Siemens AG, Erlangen, Germany) using a saturation prepared TFL product sequence with the following parameters: TR/TE=331/1.4ms ; Flip angle = 12°; BW = 668Hz/Px; Voxel size=0.8×0.8×10.0 mm (inplane interpolated); FOV=290mm2; Matrix = 170x192; Slice thickness=10mm; Acceleration=2; Inversion time= 180ms; Phase oversampling=60%; Motion correction.

Pharmacological stress was applied using Adenosine (Adenoscan®, Sanofi-Synthelabo). Gadolinium-based contrast agent (Magnevist, Bayer Schering Pharma) was administered and MRI was performed over 4 slices in short-axis orientation in breath-hold.

Image reconstruction was run twice online at the scanner: 1) with the product image reconstruction and 2) a regularized SENSE-type iterative reconstruction [4] (60 iterations, regularization 0.008). Apparent signal-to-noise ratios (aSNR) [5] were calculated in multiple regions of interest in the left ventricle and myocardium. In addition, normalized semi-quantitative upslope curves were qualitatively compared for both methods.

## Results

**Figure 1:**
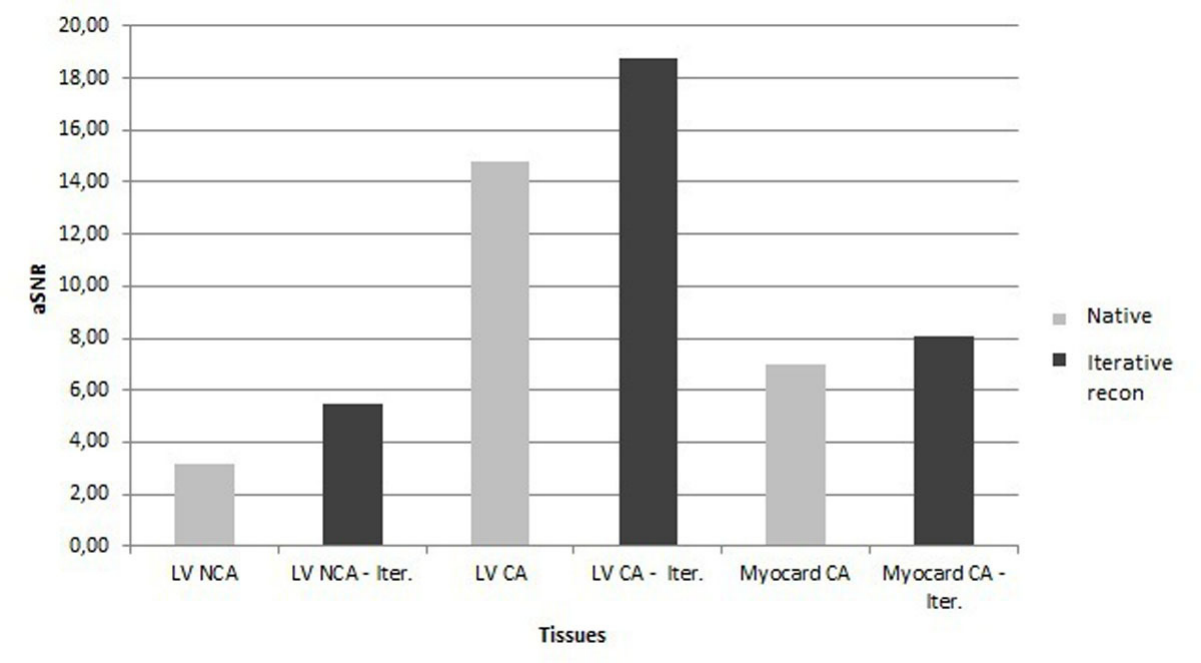
aSNR in different tissues (myocardium and left ventricle cavity without (NCA) and with Contrast Agent (CA)). Product vs. iterative reconstruction.

**Figure 2:**
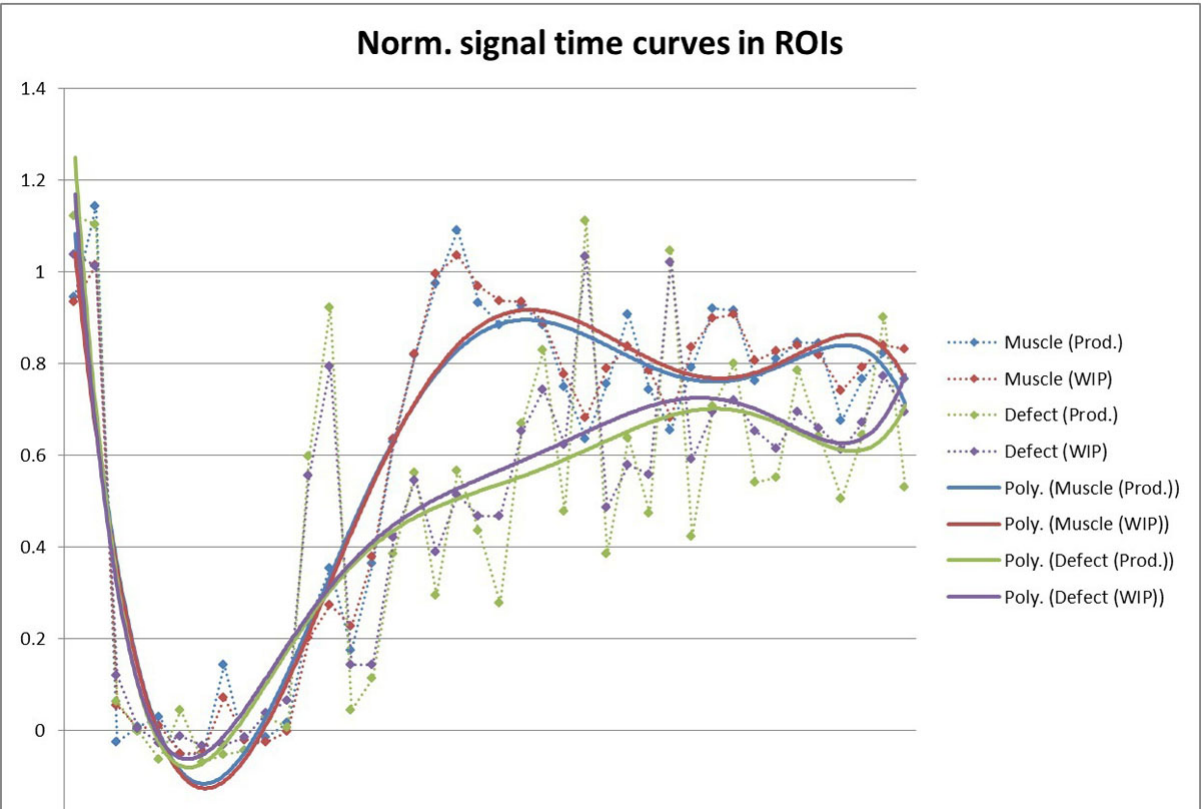
Example of signal uptake in selected ROIs with iterative (WIP) and reference (Prod.) reconstruction.

The quantitative results of aSNR in Figure 1 confirm the overall improvement of the image quality over all patients. Temporal filtering introduced by the regularized reconstruction seems to be negligible as the semi-quantitative upslope curves showed generally good concordance. Figure 2.

## Conclusions

Iterative image reconstruction shows great image quality improvements over the conventional reconstruction. In the future, SNR improvements can be invested in increasing the spatial resolution which might help to better avoid e.g. the dark rim artifact.

## Funding

None.
